# Links between prey assemblages and poison frog toxins: A landscape ecology approach to assess how biotic interactions affect species phenotypes

**DOI:** 10.1002/ece3.5867

**Published:** 2019-11-21

**Authors:** Ivan Prates, Andrea Paz, Jason L. Brown, Ana C. Carnaval

**Affiliations:** ^1^ Department of Vertebrate Zoology National Museum of Natural History Smithsonian Institution Washington DC USA; ^2^ Department of Biology City College of New York, and Graduate Center City University of New York New York NY USA; ^3^ Cooperative Wildlife Research Laboratory & The Center for Ecology Southern Illinois University Carbondale IL USA

**Keywords:** alkaloid, ant, chemical ecology, dendrobatidae, eco‐evolutionary dynamics, generalized dissimilarity modeling, multiple matrix regression with randomization, *Oophaga pumilio*

## Abstract

Ecological studies of species pairs showed that biotic interactions promote phenotypic change and eco‐evolutionary feedbacks. However, it is unclear how phenotypes respond to synergistic interactions with multiple taxa. We investigate whether interactions with multiple prey species explain spatially structured variation in the skin toxins of the neotropical poison frog *Oophaga pumilio*. Specifically, we assess how dissimilarity (i.e., beta diversity) of alkaloid‐bearing arthropod prey assemblages (68 ant species) and evolutionary divergence between frog populations (from a neutral genetic marker) contribute to frog poison dissimilarity (toxin profiles composed of 230 different lipophilic alkaloids sampled from 934 frogs at 46 sites). We find that models that incorporate spatial turnover in the composition of ant assemblages explain part of the frog alkaloid variation, and we infer unique alkaloid combinations across the range of *O. pumilio*. Moreover, we find that alkaloid variation increases weakly with the evolutionary divergence between frog populations. Our results pose two hypotheses: First, the distribution of only a few prey species may explain most of the geographic variation in poison frog alkaloids; second, different codistributed prey species may be redundant alkaloid sources. The analytical framework proposed here can be extended to other multitrophic systems, coevolutionary mosaics, microbial assemblages, and ecosystem services.

## Graphical abstract

We investigate how interactions with multiple prey species contribute to spatially structured variation in the skin alkaloid toxins of the neotropical poison frog *Oophaga pumilio*. We find that spatial turnover in the composition of ant assemblages explains part of the frog alkaloid variation, and we infer unique alkaloid combinations across the range of *O. pumilio*. Moreover, we find that alkaloid variation increases weakly with the evolutionary divergence between frog populations.

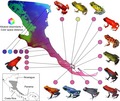

## 1 INTRODUCTION

Phenotypic variation within species, an essential component of evolutionary theory, has received increased attention by ecologists (Bolnick et al., 2011; Vindenes & Langangen, 2015). This interest has been chiefly motivated by evidence that phenotypic change, both adaptive and plastic, can happen within contemporary time scales and thus has consequences for ecological processes (Hendry, 2015; Schoener, 2011). Changes in trait frequencies can affect survival and reproduction and ultimately determine population density and persistence of a given species. In turn, these demographic changes can influence community‐level and ecosystem functions such as nutrient cycling, decomposition, and primary productivity (Miner, Sultan, Morgan, Padilla, & Relyea, 2005; Pelletier, Garant, & Hendry, 2009; Post & Palkovacs, 2009). This interplay between evolutionary and ecological processes, or “eco‐evolutionary dynamics,” has brought phenotypes to the center of ecological research (Hendry, 2015).

Several studies focusing on interacting species pairs have shown that population‐level phenotypic change can originate from biotic interactions, leading to geographic trait variation within species. For instance, different densities of Killifish predators in streams lead to distinct morphological and life‐history traits in their Trinidadian guppy prey (Endler, 1995). In western North America, levels of tetrodotoxin resistance in garter snake predators can match toxicity levels in local populations of tetrodotoxin‐defended newt prey (Brodie, Ridenhour, & Brodie, 2002). By focusing on associations between two species, these studies have provided crucial insights into how interactions can lead to trait divergence and potentially shift the evolutionary trajectory of natural populations (Hendry, 2015; Post & Palkovacs, 2009). The resulting trait diversity can have broad ecological consequences, altering the role of species in the ecosystem at a local scale (Palkovacs et al., 2009). However, we still have a limited understanding of how interactions between multiple codistributed organisms contribute to complex phenotypes, particularly when the set of interacting species and their phenotypes vary geographically.

An example of a complex phenotype that is shaped by synergistic interactions with many species is the chemical defense system of poison frogs (Dendrobatidae). In this clade of neotropical amphibians, species can exhibit dozens to hundreds of distinct lipophilic alkaloid toxins in their skin, and aposematic color patterns advertise their distastefulness (Santos, Tarvin, & O'Connell, 2016; Saporito, Donnelly, Spande, & Garraffo, 2012). Poison composition varies spatially within species such that populations closer in geography tend to have alkaloid profiles more similar to one another than to populations farther away (Mebs et al., 2008; Saporito, Donnelly, Garraffo, Spande, & Daly, 2006; Saporito, Donnelly, Jain, et al., 2007; Stuckert, Saporito, Venegas, & Summers, 2014). This geographic variation in toxin profiles has been attributed to local differences in prey availability because poison frogs obtain their defensive alkaloids from dietary arthropods (Daly et al., 2000; Jones et al., 2012; Saporito, Donnelly, Norton, et al., 2007; Saporito et al., 2004; Saporito, Spande, Garraffo, & Donnelly, 2009). For instance, specific alkaloids in the skin of a frog may match those of the arthropods sampled from its gut (McGugan et al., 2016). However, individual alkaloids may be locally present in frog skins but absent from the arthropods they consume, and vice‐versa; thus, the extent to which frog chemical traits reflect arthropods assemblages is unclear (Daly et al., 2000, 2002; Jones et al., 2012; Saporito, Donnelly, Norton, et al., 2007). Population differences may also stem from an effect evolutionary divergence, because alkaloid sequestration may be partially under genetic control in poison frogs (Daly et al., 2003; Daly, Spande, & Garraffo, 2005; Daly, Ware, Saporito, Spande, & Garraffo, 2009). These drivers of toxin variation can have broad consequences for the ecology of poison frogs, because alkaloids protect these amphibians from predators (Darst & Cummings, 2006; Gray & Kaiser, 2010; Murray, Bolton, Berg, & Saporito, 2016; Weldon et al., 2013), ectoparasites (Weldon, Kramer, Gordon, Spande, & Daly, 2006), and pathogenic microorganisms (Macfoy et al., 2005).

To address the question of how interactions between multiple organisms contribute to spatially structured phenotypes, we investigate whether prey assemblage composition turnover and evolutionary divergence between populations explain the rich spectrum of toxins secreted by poison frogs. We focus on the well‐studied alkaloid profiles of the strawberry poison frog, *Oophaga pumilio*, which exhibits over 230 distinct alkaloids over its Central American range (Daly et al., 2002; Daly, Myers, & Whittaker, 1987; Saporito et al., 2006; Saporito, Donnelly, Jain, et al., 2007). First, we develop correlative models that approximate the spatial distribution of alkaloid‐bearing ants, a crucial source of alkaloids for poison frogs (Santos et al., 2016; Saporito et al., 2012). Then, we apply generalized dissimilarity modeling (GDM) (Ferrier, Manion, Elith, & Richardson, 2007) to test whether the spatial variation in ant assemblages (i.e., beta diversity) explains chemical trait dissimilarity between sites that were screened for amphibian alkaloids, thus treating these alkaloids as a “community of traits.” To examine the effects of evolutionary divergence, we implement a Multiple Matrix Regression approach (MMRR) (Wang, 2013) that incorporates not only prey composition dissimilarity but also genetic divergences between *O. pumilio* populations as inferred from a neutral genetic marker.

## 2 MATERIAL AND METHODS

### 2.1 Estimating frog poison composition dissimilarity

**Figure 1:**
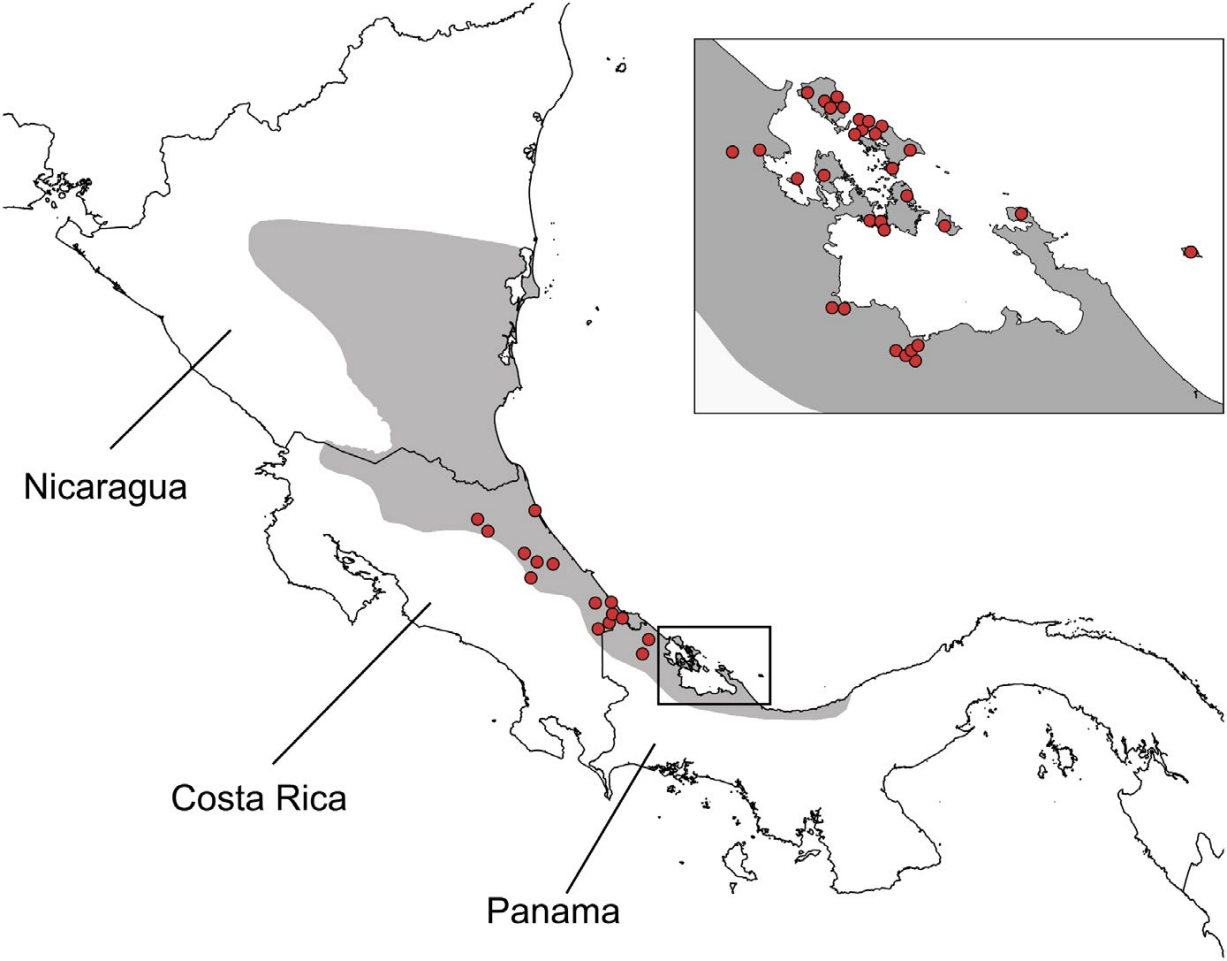
Sites sampled for skin alkaloid profiles of the strawberry poison frog, *Oophaga pumilio*. Each site corresponds to a 1 km^2^ grid cell, matching the resolution of environmental predictors. Original alkaloid data compiled by Saporito, Donnelly, Jain, et al. (2007). The distribution of *O. pumilio* is indicated in dark gray

As poison composition data of *O. pumilio*, we used lipophilic alkaloid profiles derived from gas chromatography coupled with mass spectrometry on 934 frog skins sampled in 53 Central American sites (Daly et al., 1987, 2002; Saporito et al., 2006; Saporito, Donnelly, Jain, et al., 2007), as compiled by Saporito, Donnelly, Jain, et al. (2007). We georeferenced sampled sites based on maps and localities presented by Saporito, Donnelly, Jain, et al. (2007) and other studies of *O. pumilio* (Brown, Maan, Cummings, & Summers, 2010; Gehara, Summers, & Brown, 2013; Hauswaldt, Ludewig, Vences, & Pröhl, 2011; Saporito et al., 2006; Wang & Shaffer, 2008). Because haphazard sampling may exaggerate poison composition variation in this dataset, and to maximize alkaloid sampling effort, we combined data from different expeditions to each site, pooling data from individuals. As such, we do not focus on potential short‐term individual fluctuations in toxin composition (e.g., Saporito et al., 2006), but instead on variation tied to spatial gradients. To match the finest resolution available for the data grid predictors (see below), we combined alkaloid data within a 1 km^2^ grid cell. The final dataset included 230 alkaloids from 21 structural classes in 46 grid cell sites (Figure 1) (alkaloid and locality data presented in Table S1; See Text S1 for decisions on alkaloid identity). To estimate matrices of alkaloid composition dissimilarity (pairwise Sorensen's distances) across frog populations, as well as geographic distances between sites, we used the *fossil* package (Vavrek, 2011) in R v. 3.3.3 (R Development Core Team, 2018).

### 2.2 Estimating arthropod prey assemblage dissimilarity

To approximate the spatial turnover of prey species available to *O. pumilio*, we focused on alkaloid‐bearing ants that occur in (but are not necessarily restricted to) Costa Rica, Nicaragua and Panama, where that frog occurs. Ants are *O. pumilio's* primary prey type, corresponding to more than half of the ingested volume (Caldwell, 1996; Darst, Menéndez‐Guerrero, Coloma, & Cannatella, 2004; Donnelly, 1991). This frog also eats a large proportion of mites; however, limited occurrence data and taxonomic knowledge for mite species (e.g., McGugan et al., 2016) precluded their inclusion in our spatial analyses. Following a comprehensive literature search (as per late 2016) of alkaloid occurrence in ant taxa (Adams et al., 2012; Chen, Rashid, Feng, Zhao, & Oi, 2013; Clark, Raxworthy, Rakotomalala, Sierwald, & Fisher, 2005; Daly et al., 1994, 2000, 2005; Fox et al., 2012; Jones et al., 1996, 1999, 2007, 2012; Jones, Blum, Andersen, Fales, & Escoubas, 1988; Jones, Blum & Fales, 1982; Jones, Blum, Howard, et al., 1982; Leclercq, Braekman, Daloze, & Pasteels, 2000; Ritter, Rotgans, Talman, Verwiel, & Stein, 1973; Saporito et al., 2004, 2009; Schröder et al., 1996; Spande et al., 1999; Touchard et al., 2016; Wheeler, Olubajo, Storm, & Duffield, 1981), we estimated ant composition dissimilarity based on the distribution of ant species from 10 genera, as follows: *Acromyrmex*, *Anochetus*, *Aphaenogaster*, *Atta*, *Brachymyrmex*, *Megalomyrmex*, *Monomorium*, *Nylanderia*, *Solenopsis*, and *Tetramorium*. We focus on these ant genera because species in each of them are known to harbor alkaloids (see Text S1 for decisions on alkaloid presence in ant taxa). Six out of these 10 ant genera were found in the guts of *Oophaga* species (*Anochetus*, *Brachymyrmex*, *Nylanderia*, *Solenopsis*; McGugan et al., 2016; Moskowitz et al., 2019) or other alkaloid‐sequestering frogs (*Anochetus*, *Monomorium*, *Solenopsis*, *Tetramorium*; Clark et al., 2005; Moskowitz et al., 2018). The presence of a poison frog alkaloid class in an ant taxon was sufficient for including this taxon in our dataset, regardless of whether the same alkaloid class was also found in another arthropod group (e.g., mites). Georeferenced records were compiled from the Ant Web database as per June 2017 using the *antweb* R package (AntWeb, 2017). The search was restricted to the continental Americas between latitudes 40°N and 40°S. We retained 68 ant species that had a minimum of five unique occurrence records after spatial rarefaction (see below); the final dataset included a total of 1,417 unique records. A distribution model was performed for each of the 68 ant species individually (i.e., models were not based on species composites).

Because available ant records often did not match the locations where *O. pumilio* alkaloids were characterized, we modeled the distribution of ant species to estimate prey composition at sites with empirical frog poison data. We created a species distribution model for each ant species using MaxEnt (Phillips, Anderson, & Schapire, 2006) based on 19 bioclimatic variables from the Worldclim v. 1.4 database (Hijmans, Cameron, Parra, Jones, & Jarvis, 2005; available at http://www.worldclim.org). Before modeling, we used the *spThin* R package (Aiello‐Lammens, Boria, Radosavljevic, Vilela, & Anderson, 2015) to rarefy ant records and ensure a minimum distance of 5 km between points, thus reducing environmental bias from spatial auto‐correlation (Boria, Olson, Goodman, & Anderson, 2014; Veloz, 2009). To reduce model over‐fitting, we created a minimum convex polygon defined by a 100 km radius around the occurrence points of each species, restricting background point selection by the modeling algorithm (Anderson & Raza, 2010; Phillips et al., 2009).

To properly parameterize each individual ant species distribution model (Boria et al., 2014; Shcheglovitova & Anderson, 2013), we chose the best combination of feature class (shape of the function describing species occurrence vs. environmental predictors) and regularization multiplier (how closely a model fits known occurrence records) using the *ENMeval* R package (Muscarella et al., 2014). For species with 20 or more records, we evaluated model fit using *k*‐fold cross‐validation, which segregates training and testing points in different random bins (in this case, *k* = 5). For species with <20 records, we evaluat*e*d model fit using jackknife, a particular case of *k*‐fold cross‐validation where the number of bins (*k*) is equal to the total number of points. We evaluated model fit under five combinations of feature classes, as follows: (a) linear, (b) linear and quadratic, (c) hinge, (d) linear, quadratic, and hinge, and (e) linear, quadratic, hinge, product, and threshold. As regularization multipliers, we tested values ranging from 0.5 to 5 in 0.5 increments (Brown, 2014; Shcheglovitova & Anderson, 2013). For each model, the best parameter combination was selected using AICc. Based on this best combination, we generated a final distribution model for each ant species (parameters used in all models are presented in Table S2).

To estimate a matrix of prey assemblage composition turnover based on ant distributions, we converted species distribution models to binary maps using the 10th percentile presence threshold. We then extracted a matrix of presences and absences of each ant species at each of the 46 grid cell sites sampled for frog alkaloids using the *raster* R package (Hijmans & van Etten, 2016). Based on this presence–absence matrix (presented in Table S3), we estimated a matrix of ant composition dissimilarity across the 46 sites (pairwise Sorensen's distances) using *fossil* in R.

To visualize ant species turnover across the range of *O. pumilio*, we reduced the final dissimilarity matrix to three ordination axes by applying multidimensional scaling using the *cmdscale* function in R. Each axis was then assigned a separate RGB color (red, green, or blue) as per Brown et al. (2014). A distribution layer for *O. pumilio* was obtained from the IUCN database (available at http://www.iucn.org).

### 2.3 Modeling alkaloid composition turnover as a function of ant species turnover

To test the hypothesis that toxin composition in poison frogs vary geographically as a function of the local composition of prey species, we modeled the spatial turnover of *O. pumilio* alkaloids using a generalized dissimilarity modeling (GDM) approach (Ferrier et al., 2007). GDM is an extension of matrix regression developed to model species composition turnover across the landscape as a function of environmental predictors. Once a GDM model is fitted to available biological data (here, a matrix of alkaloid dissimilarity based on empirical *O. pumilio* toxin profiles), the compositional dissimilarity across unsampled regions can be estimated based on environmental predictors (here, geospatial surfaces of ant species distributions, available for both sites sampled for *O. pumilio* alkaloids or not). We implemented GDM following the steps of Rosauer et al. (2014) using the *GDM* R package (Manion et al., 2018).

We used the matrix of frog alkaloid dissimilarity as the dependent variable in our GDMs. As predictor variables, we initially included geographic distance and all 68 individual models of ant species distributions at a 1 km^2^ resolution. To select the combination of predictors that contributed the most to GDM models while avoiding redundant variables, we implemented a stepwise backward elimination process (Williams, Belbin, Austin, Stein, & Ferrier, 2012), as follows: First, we built a model with all predictor variables; then, variables that explained less than the arbitrary amount of 0.1% of the data deviance were removed iteratively, until only variables contributing more than 0.1% were left.

To visualize estimated alkaloid turnover throughout the range of *O. pumilio* from GDM outputs, we applied multidimensional scaling on the resulting dissimilarity matrix, following the procedure outlined above for the ant species distribution models.

### 2.4 Estimating frog population genetic divergence

To evaluate associations between alkaloid composition and evolutionary divergence between frog populations, we used the *cytochrome B* gene dataset of Hauswaldt et al. (2011), who sampled 197 *O. pumilio* individuals from 25 Central American localities. Because most sites sampled for genetic data are geographically close to sites sampled for alkaloids (Hauswaldt et al., 2011; Saporito, Donnelly, Jain, et al., 2007), we paired up alkaloid and genetic data based on Voronoi diagrams. A Voronoi diagram is a polygon whose boundaries encompass the area that is closest to a reference point relative to all other points of any other polygon (Aurenhammer, 1991). Specifically, we estimated polygons using the sites sampled for genetic data as references points. We then paired these reference points with the sites sampled for alkaloid data contained within each resulting Voronoi diagram. To estimate Voronoi diagrams, we used ArcGIS 10.3 (ESRI, Redlands). We then calculated a matrix of average uncorrected pairwise genetic distances between localities using Mega 7 (Kumar, Stecher, & Tamura, 2016). Six genetically sampled sites that had no corresponding alkaloid data were excluded from the analyses.

### 2.5 Testing associations between toxin composition, prey assemblage dissimilarity, and population genetic divergence

The methods described above generated four types of distance matrices: A matrix of frog alkaloid composition dissimilarity (estimated from empirical *O. pumilio* alkaloid profiles); a matrix of ant assemblage dissimilarity (estimated from ant species distribution models); a matrix of genetic distances between *O. pumilio* populations (estimated from the *cytochrome B* dataset); and a matrix of geographic distances between sites (in kilometers). Each matrix had 46 × 46 cells, representing pairwise comparisons between each of the 46 1‐km^2^ grid cell sites that had empirical data on *O. pumilio* alkaloids.

To test how prey composition and evolutionary divergence affect poison frog alkaloid dissimilarity, we used these distance matrices to implement Multiple Matrix Regression with Randomization (MMRR) analyses in R (Wang, 2013). First, we performed analyses considering a single predictor at a time, namely ant species composition dissimilarity or genetic distances. Then, we performed an analysis considering both matrices as predictor variables simultaneously. As response variables, we used two distinct alkaloid datasets: Individual alkaloids (*n* = 230) and alkaloid structural classes (*n* = 21).

Additionally, to account for variation in poison composition resulting from ingestion of arthropod sources other than ants (i.e., mites, beetles, millipedes; Saporito et al., 2009), we performed a second set of analyses restricted to alkaloids (*n* = 125) belonging to alkaloid classes (*n* = 9) reported to occur in ant taxa.

Lastly, we tested how geographic distances affect frog alkaloid dissimilarity, ant species dissimilarity, and genetic distances.

To assess the statistical significance of MMRR models and predictor variables, we used 10,000 permutations in each analysis.

Matrices of alkaloid composition dissimilarity, estimated ant assemblage dissimilarity, genetic distances between *O. pumilio* populations, and geographic distances between sites, as well as R scripts used in all analyses, are available online through GitHub (https://github.com/ivanprates/2019_gh_pumilio).

## 3 RESULTS

### 3.1 Toxin diversity

Compilation of toxin profiles revealed large geographic variation in the number of alkaloids that compose the poison of *O. pumilio*. The richness of individual alkaloids across sites varied between 7 and 48, while the richness of alkaloid structural classes varied between 3 and 17. Alkaloid composition turnover between sites was high; pairwise Sorensen distances ranged from 0.33 to 1 for individual alkaloids and from 0.08 to 0.86 for alkaloid structural classes (Sorensen distances vary from 0 to 1, with 0 representing identical compositions and 1 representing no composition overlap across sites). A matrix regression approach using MMRR inferred that both individual alkaloid dissimilarity (*p* < .0001; *R* ^2^ = 0.16) and alkaloid structural class dissimilarity (*p* = .003; *R* ^2^ = 0.09) weakly increase with geographic distances between sampled sites.

### 3.2 Ant composition turnover

**Figure 2:**
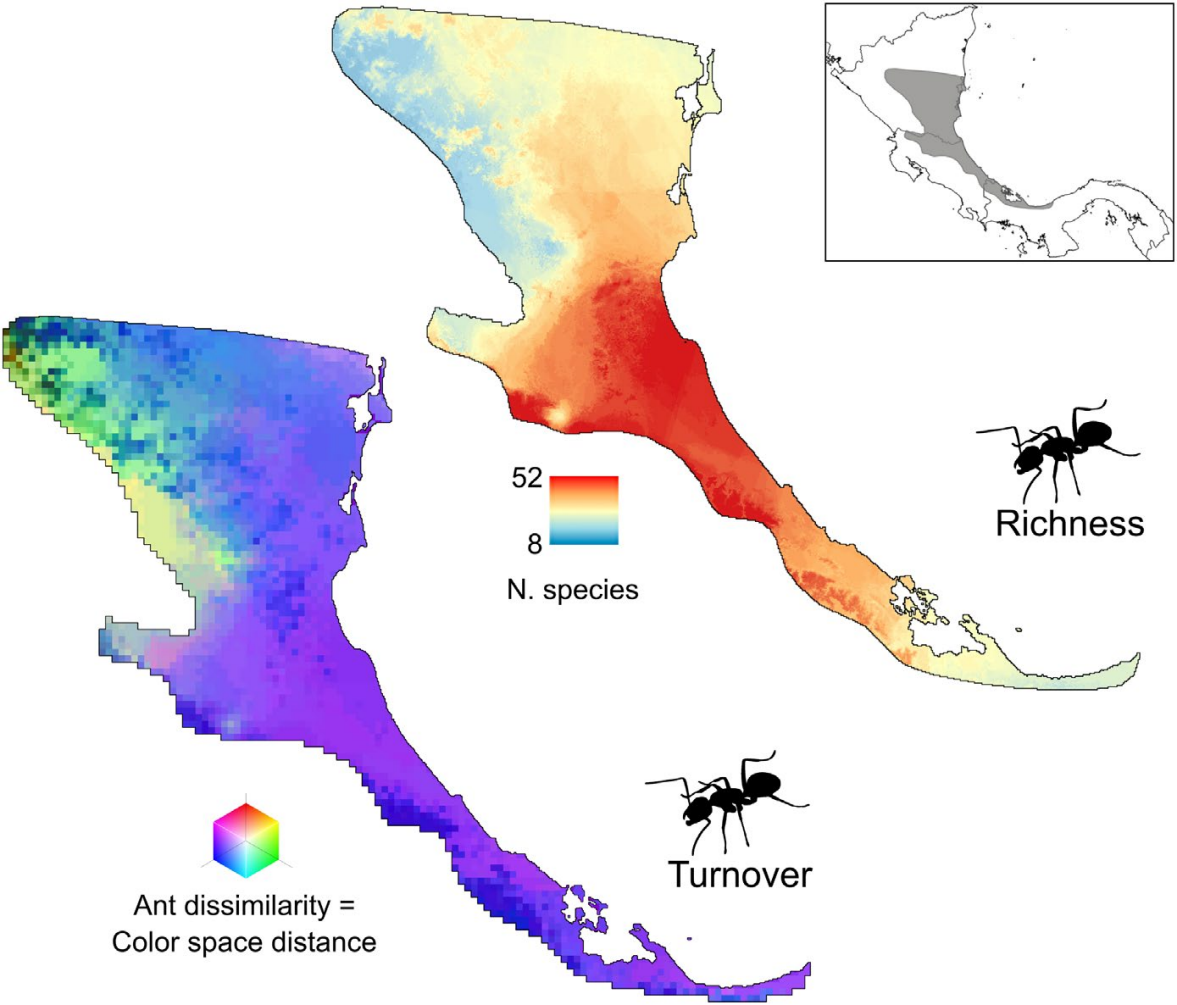
Estimated species composition turnover (left) and richness of prey assemblages across the range of the poison frog *Oophaga pumilio* based on projected distributions of 68 ant species from alkaloid‐bearing genera. Inset indicates the natural range of *O. pumilio* (dark gray)

**Figure 3:**
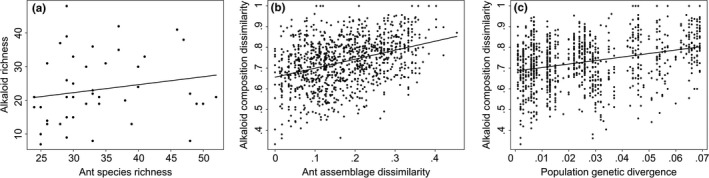
Alkaloid richness in *Oophaga pumilio* as a function of ant assemblage richness across sites (a); alkaloid dissimilarity in *O. pumilio* as a function of ant assemblage dissimilarity across sites (b); and alkaloid dissimilarity in *O. pumilio* as a function of population genetic divergence based on a neutral marker (c). Relationships in b and c are statistically significant; significance was estimated from a Multiple Matrix Regression with Randomization (MMRR) approach (see text)

Distribution modeling of ant species inferred variation in ant richness over the landscape, with a concentration of species in the central portion of the distribution of *O. pumilio* in Costa Rica and southern Nicaragua (Figure 2). The estimated number of alkaloid‐bearing ant species at sites sampled for frog toxins ranged between 24 and 52. However, ant richness did not have a significant effect on the number of individual alkaloids (linear regression; *p* > .22) (Figure 3a) or alkaloid structural classes (*p* > .07).

Ant species spatial turnover was inferred as low to moderate across the distribution of *O. pumilio* (Figure 2); pairwise ant Sorensen distances ranged from 0 to 0.47 at sites sampled for frog alkaloids. MMRR analyses suggested that dissimilarity of alkaloid‐bearing ant assemblages increases with geographic distances (*p* < .0001; *R* ^2^ = 0.62).

Projection of ant dissimilarity on geographic space suggested that prey assemblages are similar throughout the southern range of *O. pumilio* in Panama and Costa Rica, with a transition in the northern part of the range in inland Nicaragua (Figure 2). Inner mid‐elevations are expected to harbor ant assemblages that are distinct from those in the coastal lowlands.

### 3.3 Generalized dissimilarity modeling

Dissimilarity modeling supports that ant assemblage composition turnover affects the spatial variation of poison composition in *O. pumilio*. A GDM model explained 22.9% of the alkaloid composition turnover when the model included geographic distances between sampled sites. A model that did not incorporate geographic distances explained 20% of alkaloid profile dissimilarity. After eliminating those ant distribution models that contributed little to the GDM (<0.1%) using a stepwise backward elimination procedure, only nine out of 68 species were retained in the final model, as follows: *Acromyrmex coronatus*, *Anochetus orchidicola*, *Brachymyrmex coactus*, *Brachymyrmex pictus*, *Monomorium ebeninum*, *Solenopsis azteca*, *Solenopsis bicolor*, *Solenopsis pollux*, and an undescribed *Solenopsis* species (sp. “jtl001”). A GDM model not including geographic distances explained 20% of the observed frog alkaloid beta diversity also for this reduced ant dataset.

**Figure 4:**
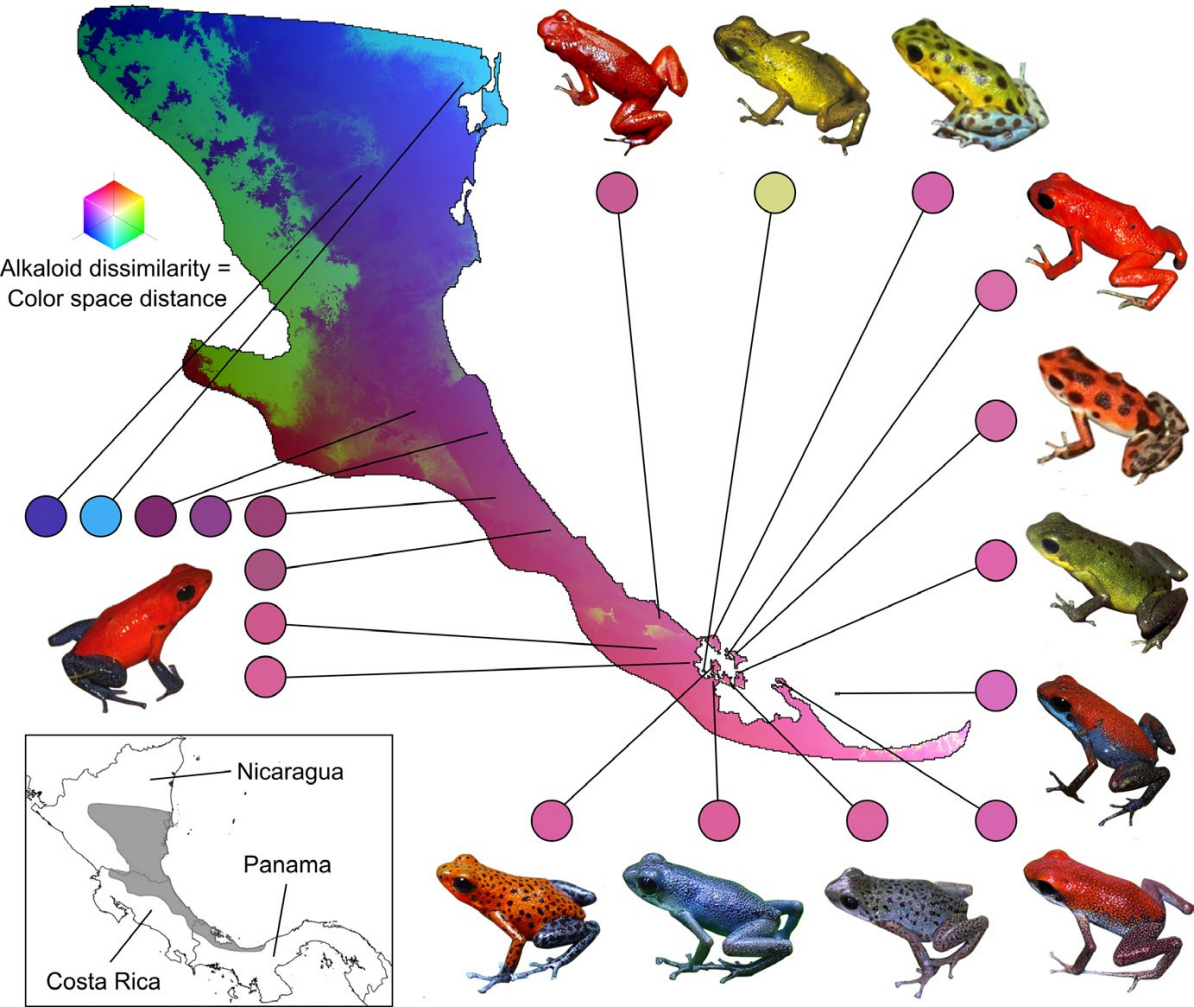
Estimated composition dissimilarity of alkaloid profiles across the range of *Oophaga pumilio* as a function of spatial turnover of alkaloid‐bearing ant species, from a generalized dissimilarity modeling approach (GDM). Similar colors on the map indicate similar estimated alkaloid profiles. Pictures indicate dorsal skin coloration patterns in *O. pumilio*. Inset indicates the natural range of *O. pumilio* (dark gray)

Projection of GDM outputs on geographic space indicates regions expected to have more similar amphibian alkaloid profiles based on ant spatial turnover (Figure 4). The results suggest latitudinal variation in poison composition, with a gradual transition from the southern part of the distribution of *O. pumilio* (in Panama) through the central and northern parts of the range (in Costa Rica and Nicaragua, respectively) (pink to purple to blue in Figure 4). Another major transition in poison composition was estimated across the eastern and western parts of the range of *O. pumilio* in Nicaragua (blue to green in Figure 4).

### 3.4 Multiple matrix regressions

In agreement with the GDM results, MMRR analyses support that alkaloid composition dissimilarity in *O. pumilio* increases with prey composition dissimilarity. Frog alkaloid composition dissimilarity increased with ant assemblage dissimilarity (*p* < .0001; *R* ^2^ = 0.13) (Figure 3b). This result changed little when considering only those alkaloids from structural classes known to occur in ants (*p* < .0001; *R* ^2^ = 0.11). Similarly, alkaloid structural class dissimilarity weakly increased with ant assemblage dissimilarity (*p* = .002; *R* ^2^ = 0.05). Considering only classes known to occur in ants yielded the same result (*p* = .007; *R* ^2^ = 0.03).

Frog alkaloid composition dissimilarity increased with genetic distances between populations of *O. pumilio* (*p* < .0001; *R* ^2^ = 0.09) (Figure 3c). Genetic distances increased with geographic distances (*p* < .0001; *R* ^2^ = 0.57), supporting that populations close in geographic space are more closely related.

An MMRR model incorporating both genetic distances and ant composition dissimilarity explained around 15% of alkaloid composition variation between populations of *O. pumilio* (*p*_ants_ < .001; *p*_gen_ = .05).

## 4 DISCUSSION

### 4.1 Effects of prey turnover on trait variation and its ecological consequences

Based on estimates of the distribution of alkaloid‐bearing ants, we found associations between prey composition variation and spatial turnover in the defensive chemical traits of the strawberry poison frog, *O. pumilio*. Species distribution modeling supported that the pool of alkaloid sources varies in space. Moreover, GDM and MMRR analyses inferred that alkaloid dissimilarity between *O. pumilio* populations increases with ant assemblage dissimilarity. These results are consistent with observations that distinct toxins are restricted to specific arthropod taxa (Saporito, Donnelly, Jain, et al., 2007; Saporito et al., 2012); prey species with limited distributions may contribute to unique defensive phenotypes in different parts of the range of poison frog species. Accordingly, GDM predicted unique alkaloid combinations in the northern range of *O. pumilio* (Nicaragua) relative to central and southern areas (Costa Rica and Panama) (Figure 4). Chemical diversity in that northern region is understudied (Mebs et al., 2008), and future surveys may reveal novel toxin combinations in the poison frogs that occur therein. These results support that biotic interactions play a role in phenotypic variation within species and across space and influence functional diversity in ecological communities (Miner et al., 2005; Pelletier et al., 2009; Post & Palkovacs, 2009).

The analyses also support that different prey species may contribute less or more to chemical trait variation in poison frogs. After eliminating those ants that contributed little to the GDM (<0.1%), only nine out of 68 species were retained in the final model. These nine species belong to five genera, of which four (*Anochetus*, *Brachymyrmex*, *Monomorium*, and *Solenopsis*) are known to be eaten by *Oophaga* or other alkaloid‐sequestering frogs (Clark et al., 2005; McGugan et al., 2016; Moskowitz et al., 2018, 2019). Therefore, a few prey items (i.e., ant species) may contribute disproportionately to the uniqueness of chemical defenses among populations of *O. pumilio*. Additionally, if different ant species provide the same alkaloids to poison frogs, an ant species may have contributed little to the GDM model in the presence of a codistributed species that has the same alkaloids; that species may thus have been removed during the GDM's stepwise backward elimination procedure. Our results pose two hypotheses: First, the distribution of only a few prey species may explain most of the geographic variation in poison frog alkaloids; second, different codistributed prey species may be redundant alkaloid sources. The second hypothesis is supported by a recent analysis of the ants eaten by a different *Oophaga* species, *O. sylvatica* (Moskowitz et al., 2019).

Our results have potential implications for the ecology and evolution of the interacting species. For instance, frogs may favor and seek prey types that provide unique chemicals or chemical combinations, increasing survival rates from encounters with their predators. This idea is consistent with evidence that alkaloid quantity, type, and richness result in differences in the perceived palatability of poison frogs to predators (Bolton, Dickerson, & Saporito, 2017; Murray et al., 2016). Moreover, because alkaloids vary from mildly unpalatable to lethally toxic (Daly et al., 2005; Santos et al., 2016), the composition of amphibian poisons may affect the survival and behavior of their predators following an attack (Darst & Cummings, 2006). From the perspective of arthropod prey, predator feeding preferences and foraging behavior may affect population densities and dynamics, potentially in a predator density‐dependent fashion (Bolnick et al., 2011; Pelletier et al., 2009). Finally, functional redundancy between prey types may confer resilience to the defensive phenotypes of poison frogs, ensuring continued protection from predators despite fluctuations in prey availability. Future investigations of these topics will advance our understanding of how the phenotypic outcomes of species interactions affect ecological processes.

### 4.2 Evolutionary divergence and phenotypic similarity

In addition to the contribution of prey assemblages, the results support that phenotypic similarity between populations increases with evolutionary divergence. MMRR analyses inferred that alkaloid beta diversity increases with population genetic distances in *O. pumilio*. This pattern may reflect physiological or behavioral differences; for instance, feeding experiments support that poison frog species differ in their capacity for lipophilic alkaloid sequestration and that at least a few species can perform metabolic modification of ingested alkaloids (Daly et al., 2003, 2005, 2009). Additionally, an effect of evolutionary divergence on toxin profiles may stem from population differences in foraging strategies or behavioral prey choice (Daly et al., 2000; Saporito, Donnelly, Jain, et al., 2007). Importantly, however, MMRR suggested that genetic divergence accounts for less variation in the defensive phenotypes of *O. pumilio* than alkaloid‐bearing prey.

We employed genetic distances from a neutral marker as a proxy for population evolutionary divergence and do not imply that this particular locus contributes to variation in frog physiology. An assessment of how genetic variants influence chemical trait diversity in poison frogs will rely on sampling of functional genomic variation, for which the development of genomic resources (e.g., Rogers et al., 2018) will be essential. Nevertheless, our approach can be extended to other systems where information on both neutral and functional genetic variation is available. These systems include, for instance, predators where nucleotide substitutions in ion channel genes confer resistance to toxic prey at a local scale (Feldman, Brodie, Brodie, & Pfrender, 2010) and species where variation in major histocompatibility complex loci drives resistance to pathogens locally (Savage & Zamudio, 2016).

### 4.3 Other sources of phenotypic variation

Although we found that variation in prey community composition may explain part of the spatial variation in frog poisons, this proportion was limited. This result may reflect challenges to quantifying spatial turnover of prey species that act as alkaloid sources. Due to restricted taxonomic and distribution information, we estimated species distribution models only for a set of well‐sampled ant species and were unable to include other critical sources of dietary alkaloids, particularly mites (McGugan et al., 2016; Saporito et al., 2012). Although not being able to incorporate a substantial fraction of the prey assemblages expected to influence poison composition in *O. pumilio*, the GDM explained about 23% of poison variation in this species. This proportion is likely to increase with the inclusion of additional species of ants and other alkaloid‐bearing prey taxa, such as mites, beetles, and millipedes (Daly et al., 2000; Dumbacher et al., 2004; Saporito, Donnelly, Hoffman, Garraffo, & Daly, 2003).

Limited knowledge of arthropod chemical diversity may also have affected the analyses. Contrary to our expectations, we found a slight decrease in the explanatory capacity of models that incorporated only alkaloids from structural classes currently known to occur in ants. This result may reflect an underestimation of ant chemical diversity. For instance, mites and beetles are thought to be the source of tricyclics to neotropical poison frogs, but the discovery of these alkaloids in African Myrmicinae ants suggests that they may also occur in ants from other regions (Schröder et al., 1996). As studies keep describing naturally occurring alkaloids, we may be far from a complete picture of chemical trait diversity in arthropods and amphibians (Saporito et al., 2012).

This study suggests that incorporating species interactions can provide new insights into the drivers of phenotypic diversity even when other potential sources of variation are not fully understood. Frog poison composition may respond not only to prey availability but also to alkaloid profiles in prey since toxins can vary within arthropod species (Dall'Aglio‐Holvorcem, Benson, Gilbert, Trager, & Trigo, 2009; Daly et al., 2002; Fox et al., 2012; Saporito, Donnelly, Norton, et al., 2007; Saporito et al., 2004). Arthropods can synthesize alkaloids endogenously (Camarano, González, & Rossini, 2012; Haulotte, Laurent, & Braekman, 2012; Leclercq, Braekman, Daloze, Pasteels, & Meer, 1996), but they might also sequester toxins from plants, fungi, and symbiotic microorganisms (Santos et al., 2016; Saporito et al., 2012). Accounting for these other causes of variation will be a challenging task to chemical ecologists. An alternative to bypass knowledge gaps on the taxonomy, distribution, and physiology of these potential toxin sources may be to focus on their environmental correlates. An extension of our approach may be to develop models that incorporate abiotic predictors such as climate and soil variation, similar to investigations of community composition turnover at the level of species (Ferrier et al., 2007; Zamborlini‐Saiter, Brown, Thomas, Oliveira‐Filho, & Carnaval, 2016).

### 4.4 Eco‐evolutionary feedbacks

Associations between prey assemblage composition and predator phenotype provide opportunities for eco‐evolutionary feedbacks, the bidirectional effects between ecological and evolutionary processes (Post & Palkovacs, 2009). In the case of poison frogs, toxicity may correlate with the conspicuousness of skin color patterns (Maan & Cummings, 2011), which therefore act as aposematic signals for predators (Hegna et al., 2013). However, coloration patterns also mediate assortative mating in poison frogs (Crothers & Cummings, 2013; Maan & Cummings, 2009). When toxicity decreases, the intensity of selection for aposematism also decreases, and mate choice may become a more important driver of color pattern evolution than predation (Summers, Symula, Clough, & Cronin, 1999). Divergence due to sexual selection may happen quickly when effective population sizes are small and population gene flow is limited, which is the case in *O. pumilio* (Gehara et al., 2013). By affecting chemical defenses, it may be that spatially structured prey assemblages have contributed to the vast diversity of color patterns seen in poison frogs. On the other hand, our GDM approach inferred similar alkaloid profiles between populations of *O. pumilio* that have distinct coloration patterns (Figure 4). However, it may be challenging to predict frog toxicity from chemical profiles (Daly et al., 2005; Weldon, 2017); future studies of this topic will advance our understanding of the evolutionary consequences of poison composition variation.

### 4.5 Concluding remarks

Integrative approaches have shown how strong associations between species can lead to tight covariation among species traits, with an iconic example being that of “coevolutionary arms races” between predator and prey species (Thompson, 2005). However, it may be harder to assess the outcomes of weaker interactions between multiple codistributed organisms (Anderson, 2017). The chemical defense system of poison frogs responds to prey assemblages composed of tens of arthropod species, each of them potentially providing only a few toxin molecules (Daly et al., 2005). Therefore, it has been hard to predict the combinations of traits emerging from these interactions (Santos et al., 2016). The landscape ecology framework presented here approaches this problem by incorporating data on biotic interactions throughout the range of a focal species, a strategy that has also improved correlative models of species distributions (Lewis et al., 2017; Sanín & Anderson, 2018). Our framework can be extended to a range of systems that have similar structures, including other multitrophic interactions (Del‐Claro, 2004; Scherber et al., 2010; Van der Putten, Vet, Harvey, & Wäckers, 2001), geographic coevolutionary mosaics (Greene & McDiarmid, 1981; Mallet & Gilbert, 1995; Symula, Schulte, & Summers, 2001), microbial assemblages (Landesman, Nelson, & Fitzpatrick, 2014; Zomorrodi & Maranas, 2012), and ecosystem services (Allison, 2012; Gossner et al., 2016; Moorhead & Sinsabaugh, 2006). Integrative studies of how spatially structured biotic interactions contribute to phenotypic diversity will continue to advance our understanding of the interplay between ecological and evolutionary processes.

## CONFLICT OF INTEREST

None declared.

## AUTHOR CONTRIBUTIONS

AC acquired funding and supervised the research team. IP, AP, and JLB designed and performed the analyses, wrote software, and worked on the visualization of results. IP obtained and curated the data and led the writing of the original draft. All four authors conceptualized the study, interpreted the results, and contributed to manuscript writing.

## Data Availability

Alkaloid and ant data at sites sampled for *Oophaga pumilio* alkaloids, dissimilarity matrices, and Supporting Information are available online through Dryad (https://doi.org/10.5061/dryad.xwdbrv193) and GitHub (https://github.com/ivanprates/2019_gh_pumilio). R scripts used in all analyses are provided in GitHub.
