# Supplementary material for: Links between prey assemblages and poison frog toxins: A landscape ecology approach to assess how biotic interactions affect species phenotypes
Source: Ecol Evol. 2019 Nov 21;9(24):14317–29. doi: 10.1002/ece3.5867 (PMC6953698; doi:10.1002/ece3.5867)
Supplement: Supplementary file 2 [file ECE3-9-14317-s002.pdf]

**Table S2.** Optimized parameters used in species distribution modeling of ant species.

| Species | N points | Feature classes | RM | Train AUC | Test AUC | OR10 | N parameters |
|---|---|---|---|---|---|---|---|
| *Acromyrmex coronatus* | 29 | LQH | 4 | 0.95 | 0.93 | 0.07 | 5 |
| *Acromyrmex echinatior* | 29 | H | 3 | 0.73 | 0.67 | 0.03 | 3 |
| *Acromyrmex volcanus* | 17 | LQH | 2 | 0.91 | 0.88 | 0.06 | 7 |
| *Anochetus diegensis* | 6 | L | 2 | 0.50 | 0.36 | 0.33 | 0 |
| *Anochetus* sp. jtl001 | 7 | L | 2 | 0.87 | 0.88 | 0.14 | 1 |
| *Anochetus mayri* | 45 | LQH | 4 | 0.83 | 0.77 | 0.02 | 7 |
| *Anochetus minans* | 5 | H | 2.5 | 0.82 | 0.80 | 0.17 | 1 |
| *Aphaenogaster araneoides* | 39 | L | 1.5 | 0.91 | 0.89 | 0.05 | 8 |
| *Aphaenogaster phalangium* | 36 | LQHP | 3 | 0.89 | 0.80 | 0.16 | 10 |
| *Atta cephalotes* | 60 | LQHP | 3.5 | 0.83 | 0.77 | 0.07 | 14 |
| *Atta colombica* | 15 | LQ | 1 | 0.68 | 0.61 | 0.20 | 2 |
| *Atta mexicana* | 39 | L | 4 | 0.72 | 0.66 | 0.08 | 4 |
| *Brachymyrmex cavernicola* | 26 | LQH | 3 | 0.87 | 0.82 | 0.16 | 6 |
| *Brachymyrmex coactus* | 11 | H | 4 | 0.86 | 0.81 | 0.09 | 3 |
| *Brachymyrmex heeri* | 38 | LQ | 1 | 0.83 | 0.77 | 0.05 | 12 |
| Brachymyrmex sp. jtl003 | 14 | LQ | 3.5 | 0.89 | 0.85 | 0.07 | 4 |
| Brachymyrmex sp. jtl004 | 7 | H | 2.5 | 0.84 | 0.80 | 0.14 | 1 |
| Brachymyrmex sp. jtl005 | 8 | H | 4 | 0.87 | 0.86 | 0.13 | 2 |
| Brachymyrmex sp. jtl007 | 23 | L | 1.5 | 0.83 | 0.77 | 0.09 | 5 |
| *Brachymyrmex longicornis* | 5 | H | 2 | 0.75 | 0.70 | 0.20 | 1 |
| *Brachymyrmex nebulosus* | 5 | LQ | 2.5 | 0.83 | 0.61 | 0.40 | 1 |
| *Brachymyrmex obscurior* | 21 | H | 2.5 | 0.87 | 0.71 | 0.13 | 7 |
| *Brachymyrmex pictus* | 10 | L | 2 | 0.75 | 0.60 | 0.40 | 1 |
| *Brachymyrmex santschii* | 9 | LQH | 3.5 | 0.95 | 0.93 | 0.11 | 2 |
| *Megalomyrmex foreli* | 11 | LQ | 1.5 | 0.84 | 0.80 | 0.18 | 3 |
| *Megalomyrmex incisus* | 15 | H | 4 | 0.82 | 0.74 | 0.07 | 3 |
| *Megalomyrmex megadrifti* | 34 | L | 2 | 0.76 | 0.73 | 0.06 | 4 |
| *Megalomyrmex modestus* | 16 | LQ | 0.5 | 0.92 | 0.87 | 0.19 | 7 |
| *Megalomyrmex mondabora* | 5 | H | 3 | 0.84 | 0.80 | 0.20 | 1 |
| *Megalomyrmex nocarina* | 5 | L | 4 | 0.50 | 0.57 | 0.00 | 0 |
| *Megalomyrmex silvestrii* | 38 | LQH | 4 | 0.74 | 0.68 | 0.03 | 5 |
| *Megalomyrmex symmetochus* | 6 | L | 2 | 0.77 | 0.69 | 0.33 | 1 |
| *Monomorium ebeninum* | 6 | LQ | 2.5 | 0.66 | 0.63 | 0.22 | 2 |

| | | | | | | | |
|---|---|---|---|---|---|---|---|
| *Monomorium floricola* | 21 | LQH | 2.5 | 0.90 | 0.86 | 0.02 | 14 |
| *Monomorium pharaonis* | 27 | LQ | 4 | 0.79 | 0.80 | 0.05 | 1 |
| *Nylanderia austroccidua* | 21 | LQH | 4 | 0.95 | 0.92 | 0.09 | 6 |
| *Nylanderia guatemalensis* | 27 | LQH | 3.5 | 0.90 | 0.88 | 0.04 | 6 |
| *Nylanderia* sp. jtl001 | 17 | LQ | 1.5 | 0.90 | 0.84 | 0.18 | 6 |
| *Nylanderia* sp. jtl007 | 5 | H | 4 | 0.82 | 0.54 | 0.20 | 1 |
| *Nylanderia* sp. jtl010 | 7 | H | 4 | 0.77 | 0.57 | 0.00 | 1 |
| *Nylanderia* sp. jtl013 | 7 | LQ | 3 | 0.82 | 0.81 | 0.29 | 1 |
| *Nylanderia steinheili* | 26 | L | 4 | 0.88 | 0.86 | 0.08 | 4 |
| *Nylanderia vividula* | 28 | L | 1.5 | 0.66 | 0.47 | 0.29 | 3 |
| *Solenopsis azteca* | 12 | L | 2.5 | 0.57 | 0.50 | 0.17 | 1 |
| *Solenopsis bicolor* | 10 | H | 3.5 | 0.88 | 0.68 | 0.30 | 3 |
| *Solenopsis brevicornis* | 33 | H | 3.5 | 0.86 | 0.83 | 0.07 | 6 |
| *Solenopsis castor* | 21 | LQH | 2.5 | 0.84 | 0.76 | 0.10 | 6 |
| *Solenopsis geminata* | 174 | LQH | 4 | 0.86 | 0.85 | 0.01 | 17 |
| *Solenopsis* sp. jtl001 | 20 | LQ | 1.5 | 0.87 | 0.83 | 0.10 | 5 |
| *Solenopsis* sp. jtl002 | 10 | H | 4 | 0.79 | 0.75 | 0.10 | 1 |
| *Solenopsis* sp. jtl003 | 16 | LQH | 3.5 | 0.89 | 0.87 | 0.06 | 4 |
| *Solenopsis* sp. jtl007 | 27 | L | 0.5 | 0.92 | 0.91 | 0.04 | 8 |
| *Solenopsis* sp. jtl014 | 8 | H | 2 | 0.91 | 0.89 | 0.13 | 2 |
| *Solenopsis* sp. jtl021 | 17 | L | 3 | 0.50 | 0.46 | 0.12 | 0 |
| *Solenopsis* sp. jtl025 | 12 | LQ | 2 | 0.80 | 0.76 | 0.17 | 2 |
| *Solenopsis* sp. jtl027 | 9 | LQ | 2 | 0.80 | 0.81 | 0.11 | 1 |
| *Solenopsis* sp. jtl031 | 7 | LQ | 2.5 | 0.84 | 0.73 | 0.29 | 1 |
| *Solenopsis picea* | 40 | LQ | 1 | 0.87 | 0.80 | 0.05 | 12 |
| *Solenopsis pollux* | 8 | LQ | 3 | 0.80 | 0.69 | 0.38 | 2 |
| *Solenopsis pygmaea* | 39 | LQ | 0.5 | 0.87 | 0.82 | 0.05 | 14 |
| *Solenopsis striata* | 22 | LQ | 1.5 | 0.84 | 0.81 | 0.05 | 5 |
| *Solenopsis succinea* | 9 | L | 1.5 | 0.86 | 0.78 | 0.33 | 3 |
| *Solenopsis vinsoni* | 26 | LQHP | 3 | 0.85 | 0.80 | 0.15 | 10 |
| *Solenopsis zeteki* | 27 | LQH | 3 | 0.89 | 0.87 | 0.04 | 7 |
| *Tetramorium bicarinatum* | 14 | L | 1.5 | 0.87 | 0.84 | 0.20 | 3 |
| *Tetramorium lanuginosum* | 12 | L | 2.5 | 0.70 | 0.62 | 0.27 | 1 |
| *Tetramorium simillimum* | 18 | H | 2.5 | 0.83 | 0.74 | 0.33 | 3 |
