# Supplementary material for: Links between prey assemblages and poison frog toxins: A landscape ecology approach to assess how biotic interactions affect species phenotypes
Source: Ecol Evol. 2019 Nov 21;9(24):14317–29. doi: 10.1002/ece3.5867 (PMC6953698; doi:10.1002/ece3.5867)
Supplement: Supplementary file 4 [file ECE3-9-14317-s004.pdf]

Text S1. Decisions on alkaloid identity and alkaloid presence in ant taxa.

1. We considered *Aphaenogaster* ants to have alkaloids because they have anabaseine, which is analogous to the frog alkaloid noranabasamine (Daly et al., 2005).
2. Alkaloids were detected in *Paratrechina steinheili* (Saporito et al., 2004) and *P. amblyops* (Clark et al., 2005). These two species are now allocated in the genus *Nylanderia* (La Polla et al., 2010). No *Paratrechina* (as currently recognized) is included in our study.
3. *Carebarella*, found to contain HTX (Jones et al., 2012), is now within *Solenopsis* (Pacheco and Mackay, 2013).
4. There does not appear to be a deohy-PTX class, but there is a deoxy-hPTX class (Daly et al., 2005). deohy-PTX may have been listed in error by Saporito et al. (2007).
5. Alkaloids 235B, 235B', and 235B" were treated as 235B, following Daly et al. (2005).
6. Alkaloids 195A, cis-195A, and trans-195A were treated as 195A, following Daly et al. (2005).
7. Alkaloid "321", listed for a single site, was included. Although it is no clear whether it represents alkaloid 321A, B, C, D, or E, this alkaloid is not present in any other site.
8. Alkaloid "325", listed for a single site, was excluded. The reason for this exclusion is that it is not clear whether it represents alkaloids 325 A, B, or C, which are present in other sites.
9. There seems to be no 207H listed for any sampled site in the Supplementary Information of Saporito et al. (2007), even if there is in the main text. The total number of alkaloids listed by Saporito et al. (2007) therefore seems to be 231 and not 232.
10. Daly et al. (2005) report 237C (5,6,8-I) to be of ant origin, but Saporito et al. (2009) do not. Following Saporito et al. (2009), we do not treat 5,6,8-I as being of ant origin.
11. Clark et al. (2005) found alkaloid 225C in an ambiguous mixture of *Camponotus* and *Pachycondyla* ants. We conservatively do not consider them to be alkaloid-bearing ants.
12. Tricyclics were not excluded from analyses restricted to ant alkaloids because this class was found in African Myrmicinae ants (Schroder et al., 1996).
